# Remarks on Fever and Rheumatism; Case of Metastasis of Gout; and a Case of Coup De Soleil. Being Extracts from a Report of the Diseases Which Prevailed in the 2d Dragoon Guards, during the Year 1825

**Published:** 1826-03

**Authors:** Andrew Browne

**Affiliations:** Surgeon of the Regiment.


### Art. III.—*Remarks on Fever and Rheumatism; Case of Metastasis of Gout; and a Case of Coup de Soleil. Being Extracts from a Report of the Diseases which prevailed in the 2d Dragoon Guards, during the Year* 1825. By Andrew Browne, Esq. Surgeon of the Regiment.

### FEVERS.

Under this head, I include every class of disease in which the arterial action has been accelerated, and the temperature of the skin increased, preceded or ushered in by more or less languor and debility; and, as the greater portion of febrile cases of this character, treated between the end of December 1824, and the beginning of September 1825, were mild to a degree, and required but a very short period of confinement to hospital, or indeed any great degree of solicitude, I shall now pass them over without comment, and limit myself to a description of the epidemic, which first appeared amongst us about the latter period; having then changed the mild garb it formerly wore, and perceptibly assumed that of synochus.

About the latter end of August and beginning of September, our febrile admissions augmented rapidly, and their character likewise manifestly changed, having become more acute and obstinate. But, on the commencement of the continued rains in this latter month, the symptoms became highly aggravated, and continued in an increased ratio, until checked in some degree by the light nocturnal frosts in the beginning of November.

And I here consider it worthy of remark to state, that, at the commencement, or during the early part of this disease, it was principally confined to those men recently relieved from the coast-duty, and to the soldiers' rooms at the west end of the barracks: in a very short time, however, it gradually and insidiously advanced to the other extremity of the line, visiting slightly, in its passage, a few of the officers in the centre. But nearly every woman and grown child in each wing suffered severely from its attack; so that it became necessary to convert one of the attic stories of the men's barracks into an hospital for their accommodation. This I state merely as an occurrence, without attempting to advance an opinion, or even a supposition, that this fever was, in any instance, derived from the agency of contagion; although every precautionary measure that art and experience could suggest, as to cleanliness and comfort, was throughout most rigidly kept in practice. But I apprehend that both the remote and exciting causes may be safely ascribed to the late sudden transition of season; to cold, producing an increased impetus of the blood, with a determination to the abdominal viscera; to miasma; to want of natural rest and regular meals; to checked perspiration, &c.

*Symptoms.*—The leading symptoms generally complained of in this disease were, extreme depression of spirits, preceding loss of appetite; headache, lassitude, rigors; irritability of stomach, with an obtuse pain, or sense of weight, in the epigastrium, "as if of a burning coal;" with a frequent acute pain in the right hypochondrium, (often in both,) preventing the slightest contortion of the body; general soreness, and wandering pains, particularly severe over the loins and extremities; great prostration of strength; foul tongue; much thirst; a small, hard, frequent, vibrating pulse; skin dry, and considerably above the temperature of health. But the bowels frequently undisturbed, and often obstinately prone to costiveness; and, when brought into action by cathartics, the alvine excretions were invariably of a dark green colour, often black as pitch, and unusually fetid.

In many, however, the disease manifested itself, in the commencement, (as above described,) with considerable nervous irritability, which was evinced, even in the stoutest and most robust, by a copious flow of tears, previous to any answer given to our interrogatories; great prostration of strength; headache; anorexia; severe and constant pain over the whole abdomen, with soreness on pressure, and a copious purging of dark green slimy stools, having a peculiarly offensive smell, and without affording even a momentary relief; much thirst; the tongue covered, at its root, with a thick brown crust, soon spreading over its entire surface. Pulse small and quick; skin hot and dry.

In the second stage of this epidemic, the pain in the epigastric and hypochondriac regions became gradually less acute, and painful only on pressure. The alvine excretions, always of a dark green colour, became now intermixed with long white shreds. Respiration frequent and anxious; occasional nausea, and vomiting a light green, frothy, bilious matter. Skin of the palms and tendons of the wrists tense; with constant mental alienation, and partial paralysis of the lower extremities. Urine scanty and high coloured; mouth distressingly dry and clammy. Pulse more moderate, and skin more temperate; without, however, the slightest perceptible exacerbation or remission after the seventh or eighth day.

The last stage was marked by a total cessation from pain or soreness; the patient always saying "that he was better," or "that he was very well." The tongue still dry and parched, of a logwood colour, but free from its original crust; and its action nearly lost, there being much difficulty in propelling it beyond the teeth. Excretions unaltered, and now passed involuntarily. Pulse scarcely perceptible, and the skin cold and clammy. Continual picking at the nose or bedclothes; subsultus tendinum; sinking down off the bolster to the foot of the bed; hiccup; and, in one instance, vomiting a dark brown grumous matter, so often noticed by West India writers on the bilious remittent fevers of those climates.

*Post-mortem appearances.*—The diseased appearances presented on dissection being all nearly similar, or at least without any essential difference in character, I herewith transcribe one of them from my register, which may tend to illustrate the foregoing account of the symptoms.

On lifting the sternum, twenty-five hours after death, and exposing the cavities of the thorax and abdomen, there exhaled from them a peculiarly offensive odour. In the chest, the lungs were found turgid; without, however, any vestige of recent disease, although the left lobe adhered firmly to the pleura costalis,—the effect, no doubt, of former inflammatory action. The heart and pericardium, with the other contents of this cavity, healthy. But the liver appeared fuller and paler than natural, with a large livid patch on the centre of its convex surface, and a dark slate-coloured fringe, or border, two inches deep, along the whole of its inferior edge, on both concave and convex sides, and penetrating into its substance about the sixteenth part of an inch. The peritoneum over the whole anterior surfaces, interspersed with small vesicles; and, on carefully dissecting the lobes from their respective connexions, and placing them on the table, they appeared preternaturally large, and unusually soft and attenuated, incapable of resisting a moderate pressure of the finger; but nearly devoid of blood.

The gall-bladder perfectly empty, and its coats, with those of the bile-ducts, thickened and enlarged. The hepatic artery, after its entrance into the fissure of the liver, as well as the vena portæ, were likewise thickened in all their ramifications; exhibiting conclusive evidence of previous inflammation. The small lobe unusually large, but less disorganised.

The diaphragm, kidneys, and pancreas, comparatively healthy.

The spleen nearly double its natural size, resembling the liver in every particular; but, on cutting into its substance, it was found to contain much dark fluid blood.

In the stomach was found ten or twelve ounces of a dark tenacious matter, which, on being removed, the whole of its internal surface exhibited to view a number of starlike, vermilion coloured petechiæ; most numerous about the pyloric orifice.

The whole of the mesentery of a dusky claret colour, and its vessels filled with blood.

The brain and its meninges suffered, likewise, from recent inflammatory action. The vessels on the whole surface were unusually large, and distended with dark fluid blood; and about six ounces of serum were found in the ventricles. But the substance of the brain itself appeared tolerably healthy, although, perhaps, more firm than natural in its texture.

*Treatment.*—In every case admitted, there was always present more or less increased action of the sanguiferous system, and apparent congestion either in the liver or head,—generally in both. The treatment, therefore, in the commencement of this disease, was to abstract blood from the arm by a large orifice, until syncope was produced, or every vestige of the pain in the side and head ceased; the blood invariably exhibiting, a few minutes after abstraction, a buffy or inflammatory crust, peculiarly dense; and the robust and plethoric oftentimes losing *sixty-four ounces and upwards at the onset*, which very frequently had the effect of saving a repetition, and thereby consequent subsequent debility. However bold this practice may appear to many, I have long been fully confirmed in it, by observing its effects in arresting (in nine cases out of ten) the progress of the complaint at once, and thereby preventing frequent recurrences to the lancet, which often ultimately fail, and invariably hasten the debility attendant on the second stage of acute or inflammatory fevers; a circumstance very important to guard against.

The next step in the treatment was the evacuation of the contents of the stomach, by administering, shortly after the operation of blood-letting, small doses of a solution of tartarised antimony, until they produced a complete effect; with the view not only of causing sufficient evacuation, but to relieve, by its action, the congestion in the liver, to determine to the skin, and to produce a direct and emulgent effect on the other viscera.

To this end, likewise, mercurial purgatives, combined with antimony or James's powder and colocynth, were administered every four or six hours, assisted by supertartrate of potass, neutral salts, or infusion of senna, until the bowels were well evacuated, and the alvine excretions assumed a natural colour, and less offensive odour.

Blisters were also occasionally had recourse to, where the wanderings of the mind were rapid, and the temper irritable and violent. They assisted likewise in restoring the balance of the circulation; but were, in general, rendered unnecessary by previous depletion and the free use of purgatives, diaphoretics, and saline effervescing draughts.

If, however, the employment of these means did not succeed in entirely subduing the paroxysm in its first stage, and the disease ran into the second, assuming a chronic form, mercury was forthwith exhibited, both internally and externally, with the most favourable result; as, the moment its action became visible on the system, I could confidently and unerringly pronounce the patient out of danger; and in many instances, where the mercurial purges alone, exhibited in the first stage of the disease, excited slight ptyalism or fetor of the breath, every symptom of the disease instantly ceased, and the alvine excretions resumed their natural colour. But, whether owing to the peculiarity of the disease or the constitution of the patient, the system, in many, most obstinately resisted every effort to excite a mercurial action, and this was particularly observable in those cases where the admission into hospital did not take place during the first stage. A copious purging of dark green bilious matter, with irritability of stomach, then became the leading symptoms.

Under these circumstances, after the use of effervescing aperients and blisters, recourse was necessarily had to stimulants and cordials, to prevent increasing debility and threatened dissolution; and, in every case of this description, the most unfavourable prognosis must reasonably be formed, the recovery being always tedious, and often imperfect, leaving a strong disposition to chronic hepatitis; of which we have still five cases remaining in hospital, requiring some care, and an occasional mercurial purge.

But, in every description of this epidemic, whether in an early or advanced state of convalescence, I soon discovered that the exhibition of bark in any form, or indeed of any other tonic, was invariably prejudicial, reproducing headache, dryness of skin, pain in the hepatic region, and a return of dark-coloured slimy stools; while benefit was derived from a light nutritive diet, diluted port wine, and good strong Dorchester beer.

Under this plan of treatment, however severe and alarming the symptoms might have been on admission, (when this took place a day or two after the attack,) I felt perfectly easy as to the result; and, so far from inducing increased debility, too often and groundlessly dreaded in such cases, it has, on the contrary, not only procured the patient immediate relief from the severity of his sufferings, but an apparent renewal of strength and energy of system, on his perfect recovery; as, in no one instance where the early and vigorous employment of the measures herein described were had recourse to, has there been the slightest disposition to a recurrence; a result which I have long experienced in tropical as in temperate climates, on the low swampy shores of Demerara, Esequibo, Berbice, and Surinam, where heat and moisture are so powerfully combined, as on the high light soil of many of our West India islands, in Gibraltar, and now at home, where this fever is virtually the same, modified no doubt by local circumstances.

---

### CASE OF ICTUS SOLARIS.

During the unusual and excessive heats in the months of June, July, and August, we had frequent instances of men, exposed to the sun's rays, being suddenly attacked with severe headache, vertigo, and occasional irritability of stomach; which were generally removed in a day or two, by a saline purge and confinement within doors. But, on the 24th of August last, between three and four o'clock in the afternoon, when the atmosphere must have been heated to 120° of Fahrenheit, I was summoned to a field adjoining the barracks, to see a man, who was reported "to have dropped suddenly dead;" and, on hastening to the spot, I found one of our farriers, a fine, stout, plethoric, muscular man, about twenty-five years of age, extended where he fell, perfectly senseless and motionless; his limbs remaining in any position in which they were placed; his breathing, however, free and natural; his skin considerably above the temperature of health, although previously dashed with cold water, from the adjoining river, by his companions; his countenance flushed; the vessels of the eyes turgid, the pupils very much dilated, and a total want of contractile power in the iris; his pulse peculiarly full and firm, but not rapid; his jaws firmly closed, except on occasional irritability of stomach, when its contents were partially ejected, without any effort; and his fæces and urine had just passed involuntarily.

On inquiry, I soon ascertained that he had been drinking beer, but not to excess; that he had been leaping and running with his comrades in succession, for nearly two hours previously; that he had no clothes on, except his shirt and drawers, and that his head, during the whole time, was uncovered, and exposed to the rays of this unusually hot sun; and that, in the middle of his last race, when apparently winning it, he dropped suddenly, as if shot through the head.

*Treatment.*—With this information, and the general aspect of the case, I felt confident that nothing short of the most vigorous means afforded a prospect of saving his life; the first object, therefore, after his immediate removal into hospital, was to diminish the quantity of circulating fluid, and thereby lessen the tone of the vessels in the system generally, and in the head particularly. To this end, large bleedings from the arm, temporal arteries, and jugular veins, were forthwith had recourse to; saline glysters, refrigerants, cool air; and a constant erect posture was maintained, to assist in taking off the force of the blood in the vessels of the head: this was evidently and materially assisted by a shower-bath every fourth hour. By a bold continuation of these measures, in which he lost in the commencement 112 ounces of blood, some slight improvement was apparent on the 30th, six days after admission; when he first showed consciousness of surrounding objects, and a sense of tension was referred to the chest and head, by a movement of the right hand. But, at the same time, it was clearly discovered that hemiplegia, or perfect paralysis of the left side, had taken place, with the mouth considerably contorted to the right side, and the left eye totally insensible to the rays of light.

Blisters, drastic purgatives, friction, and electricity, in conjunction with the shower-bath, were now ordered, and continued with daily increasing advantage; and, although he was long after subject to severe headaches, a peculiar affection of the eyelids, the mind not readily exercised, and the vital functions somewhat impaired, yet, by a steady and unceasing application of these means, the whole of the latter symptoms were entirely removed on the 2d of October following, thirty-nine days after admission, when he was permitted to return to his barracks, to attend to his business.

He has since resumed his duty in the forge; and, although he still feels some deficiency of strength in the affected side when at hard work, yet it is now scarcely perceptible, either in walking or performing light duties; so that the recovery is complete beyond my most sanguine expectations.

RHEUMATISMUS.

Dragoons are necessarily more subject to this class of disease, from the peculiarity of their duties, than any other description of soldier, being employed at least four hours every day in grooming and cleansing their horses, in a stable heated by excrementitious vapour and animal effluvia, fifteen or sixteen degrees higher than the circumambient atmosphere, to which they often, within this period, suddenly expose themselves (although contrary to order,) to draw water from the pump, frequently without their jackets, and always in a greater or lesser degree of perspiration; and, consequently, there are but very few of the old soldiers of the corps entirely exempt from this disease.

The greater part, however, of the individuals admitted under this head, when placed under treatment, were without any febrile or acute symptoms; complaining of erratic pains in almost every part of the body, but particularly severe in the large joints, without redness or swelling, and always augmented by warmth in bed; with evening exacerbations. Functions generally regular, and appetite always tolerably good; yet, after a short period of suffering, the bodily strength was diminished, the limbs and muscles wasted, and a peculiar haggard appearance of the countenance followed, always influenced more or less by change of weather; and distinctly separate from any connexion with either syphilis, scurvy, or the previous use or abuse of mercury.

*Treatment.*—In the very great variety of treatment heretofore tried in this tormenting disease, all external applications appeared to me invariably attended with but a momentary good effect,—removing, perhaps, the pain from the part affected to drive it to another. The internal use of colchicum, in increasing doses, had decidedly the preference over the whole of them. Acupuncturation, as lately recommended by Churchill, has been tried nearly upon them all, and on some few with a transitory good effect. But, when there is no apparent phlogistic diathesis present, I have lately found that mercurial vapour, applied to the whole surface night and morning, until a slight action is perceptible, has an unequivocal advantage over every other remedy I have yet tried; giving tone and activity to the circulating system, requiring no muscular exertion in its use, being cleanly and safe in its application: and, whether the complaint lies in the muscular fibre, in the ligaments, or in the coats of the arteries themselves, I cannot hesitate in recommending this active diaphoretic and alterative as, at least, a powerful auxiliary.

### METASTASIS OF GOUT, FROM THE EXTREMITIES TO THE ABDOMINAL AND THORACIC VISCERA.

This disease, so unusually met with in military practice, was long a frequent companion of our late quarter-master, a man of robust and plethoric habit, a cholerico-sanguine temperament, fond of good living, with a full florid complexion, and about fifty-two years of age. Having been long subject to attacks of it in a regular form, previous to my joining the Bays, I cannot, in consequence, enter fully into its previous history from personal observation; but it appeared from his own statement that he was, early in life, a martyr to severe attacks of rheumatism, both acute and chronic, which almost imperceptibly ran into regular gout about seven years since, suffering slight autumnal paroxysms, until the month of October, 1823, when a fit was repelled by some nostrum, which he was fond of resorting to, and was forthwith succeeded by acute pain in the præcordia; the stomach, so universally in consent with the rest of the system, becoming suddenly and violently affected; the lungs and heart participating; accompanied by a spasmodic affection in the epigastric region, severe vomiting, great anxiety, difficult respiration, palpitation, syncope, orthopnea, and laborious arterial action; which were ultimately mitigated, and with great difficulty subdued in six days, by copious blood-letting, warm baths, antispasmodics, blisters, opiates, musk, squills, and camphor.

But, although the high inflammatory action was at the time subdued, the effects of his imprudence continued to remain, without the slightest symptom of his former periodical gout ever after returning; and, in the autumn of 1824, twelve months after the attack above described, the following symptoms were noted in my private journal:—

A constant, dry, irritable cough, with a copious expectoration of a thick viscid phlegm; a sighing, suffocating respiration, rendering it necessary for him to be always erect to breathe freely. Loathing of food, and partial loss of appetite; without, however, any well-defined febrile symptom. Pulse full, but elastic and easily compressed, at ninety-two, with intermissions at every tenth or twelfth pulsation. Vertigo on any unusual exertion, violent palpitations, and increased dyspnœa on walking up stairs. A constant obtuse pain in the left breast, increased to acute by a recumbent position; and a frequent painful constriction across the whole chest, extending around each deltoid muscle. The external form of the thorax unaltered; but a general soreness prevails all over it, so as to render percussion inadmissible. The action of the heart visibly laborious, extending over a space of six or seven inches in diameter; and the whole arterial canal corresponding or vibrating with it, even to its remotest branches.

In this state he continued, with those symptoms at times considerably less aggravated; influenced, he thought, by the state of the atmosphere, feeling better when the air was dry and elastic; but always deriving temporary relief from antispasmodics, until the month of December following, when he first formed an idea that the exposed situation of Norwich barracks was detrimental to him, and that a change during the cold months might be productive of benefit.

Under this impression, he removed to Ipswich in January last, and placed himself under the medical care of a private practitioner of that place, who appears to have tried mercury to copious salivation, demulcents, and expectorants, without the least benefit. He therefore returned to head-quarters in April following, with a very visible alteration for the worse, in his symptoms and general appearance; having become emaciated, appetite quite gone; and, in addition to all his former sufferings, harassed by frequent severe headaches, and constant distressing incubus, which now became so much augmented as to compel him to remain constantly within doors: in which state he continued until the morning of the 20th of May, when, after shaving and dressing himself as usual, he complained of general tremors and unusual weakness, which compelled him to return to bed; immediately after which he died, without the slightest convulsive struggle, rotten with mercury

*Dissection.*—On the corpse being uncovered, forty hours after death, the external form of the chest appeared materially altered, with considerable emphysema, extending from the left axilla to the hypochondrium, with some ecchymosis; and, on removing the integuments, the muscles of the trunk presented a soft, flabby, ash-coloured appearance: on being detached, they were readily mashed, or divided by simple pressure between the finger and thumb.

The ribs likewise were quite loose in their articulations with the vertebræ, offering little or no resistance to the saw, so that each rib required support to enable it to be cut through; and, at length, on lifting the sternum, the lungs appeared unusually distended with blood and air, starting above the surface of the thorax, as far as firm and extensive adhesions of each to the pleura costalis and pericardium would admit; and, on separating them from their connexions, and lifting them from their cavities, incisions into them were followed by the escape of a dark bloody mucus, intermixed with a thin purulent matter, and their substance, throughout their whole extent, studded with tubercles and concretions.

The pleura costalis, diaphragm, and præcardium, were preternaturally thick and emphysematous, and their internal surfaces throughout of a deep violet colour; not giving the idea of recent inflammatory action by the presence of ramifying vessels, but of infiltration of long standing; and in the cavity of the latter was found more than six ounces of bloody serum.

The heart itself was truly enormous, being double its natural size, and occupying a third of the whole cavity of the chest; pale and flaccid as the external muscles, and surrounded by much gristly fat. The parietes of the right auricle and ventricle much softened or attenuated, the latter being in some parts nearly transparent; the left not so thin, but equally soft and flaccid. The opening between the right auricle and ventricle unusually large. The valves distended, and the internal surface of each, with the membranes lining the cavities of the vena cava and pulmonary vessels, were universally of the same violet colour already described, but somewhat darker.

The aorta partook of the same dusky hue, and its arch was covered, for about three inches, with honey-combed or ulcerated spots, some of which had nearly penetrated its coats: hence the bloody infiltration into the pericardium already noticed.

The stomach unnaturally small, and thickened throughout its entire substance. The villous coat of a dark chocolate colour; and the longitudinal plaits, or rugæ, near the pylorus considerably raised or thickened, with the interstices filled with a caseous matter, resembling indurated or coagulated lymph.

The head was not examined; but the remaining viscera, were free from morbid appearances.

